# The interplay of depressive symptoms and self-efficacy in adolescents: a network analysis approach

**DOI:** 10.3389/fpsyg.2024.1419920

**Published:** 2024-08-30

**Authors:** Xiang Li, Bizhen Xia, Guanghui Shen, Renjie Dong, Su Xu, Lingkai Yang

**Affiliations:** ^1^Wenzhou Seventh People’s Hospital, Wenzhou, China; ^2^Qingtian County People’s Hospital, Lishui, China; ^3^Zhoupu Community Health Service Center, Shanghai, China; ^4^Department of Psychology, School of Education, Wenzhou University, Wenzhou, China

**Keywords:** self-efficacy, adolescent depressive symptoms, network analysis, LASSO, psychological constructs

## Abstract

**Background:**

Self-efficacy, a critical psychological construct representing an individual’s belief in their ability to control their motivation, behavior, and social environment. In adolescents, self-efficacy plays a crucial role in mental health, particularly concerning depressive symptoms. Despite substantial research, the complex interplay between self-efficacy and depressive symptoms in adolescents remains incompletely understood.

**Aims:**

The aim of this study is to investigate the complex interrelationships between self-efficacy and depressive symptoms in adolescents using psychological network analysis.

**Methods:**

The cross-sectional study involved 3,654 adolescents. Self-efficacy was assessed using the General Self-Efficacy Scale (GSES), and depressive symptoms were measured with the Patient Health Questionnaire-9 (PHQ-9). Network analysis, incorporating the least absolute shrinkage and selection operator (LASSO) technique and centrality analysis, constructed and compared self-efficacy networks between depressive symptoms and healthy control groups.

**Results:**

Of the 3,654 participants, 560 (15.32%) met criteria for moderate to severe depressive symptoms (PHQ-9 scores ≥10). Among those with depressive symptoms, 373 (66.61%) had moderate, 126 (22.50%) had moderate–severe, and 61 (10.89%) had severe symptoms. Bivariate correlation analyses revealed a significant negative correlation between depressive symptoms and self-efficacy (*r* = −0.41, *p* < 0.001). The results of the network analysis showed significant differences in self-efficacy networks between adolescents with and without depressive symptoms (global strength: *S* = 0.25, *p* < 0.05). Depressed participants showed a network with reduced global strength, suggesting diminished interconnectedness among self-efficacy items. Specific connections within the self-efficacy network were altered in the presence of depressive symptoms. Bridge analysis revealed that effort-based problem-solving (bridge strengths = 0.13) and suicidal ideation (bridge strengths = 0.09) were the key bridge nodes.

**Conclusion:**

Adolescent depressive symptoms significantly impacts the self-efficacy network, resulting in diminished integration of self-efficacy and highlighting the complex interplay between self-efficacy and depressive symptoms. These findings challenge the traditional unidimensional view of self-efficacy and emphasize the need for tailored interventions focusing on unique self-efficacy profiles in adolescents with depressive symptoms.

## 1 Introduction

Self-efficacy, as introduced by psychologist Albert Bandura, is an individual’s belief in their capacity to execute behaviors necessary to produce specific performance outcomes (Bandura, 1994). It reflects confidence in the ability to exert control over one’s own motivation, behavior, and social environment (Maddux, 2016). This concept has become a significant focus in psychology due to its influence on how people think, behave, and feel, especially in various challenging situations (Yıldırım and Güler, 2022). In the context of adolescent mental health, self-efficacy plays a crucial role (Kim et al., 2019). Adolescence, characterized by numerous developmental challenges, is a critical period wherein an individual’s belief in their ability to surmount obstacles can substantially impact their psychological well-being. Depression, a common and often debilitating condition among adolescents, can be profoundly influenced by levels of self-efficacy (Shorey et al., 2022). The relationship between self-efficacy and depression in adolescents is complex and bidirectional. According to Bandura’s Social Cognitive Theory, individuals with high self-efficacy are more likely to view difficult tasks as challenges to be mastered rather than threats to be avoided, leading to lower vulnerability to stress and depression (Bandura, 2001). Conversely, low self-efficacy can contribute to the development and maintenance of depressive symptoms (Calandri et al., 2021). This relationship is further elucidated by the cognitive vulnerability-stress model of depression, which posits that negative cognitive styles, including low self-efficacy, interact with stressful life events to precipitate depressive episodes (Hankin, 2008).

A large body of research supports these theoretical frameworks. A meta-analysis by Yeo et al. (2023) found a significant negative correlation between self-efficacy and depressive symptoms in children and adolescents (Yeo et al., 2023). Longitudinal studies have demonstrated that changes in self-efficacy predict changes in depressive symptoms over time. For instance, Tak et al. (2017) found that decreases in academic and social self-efficacy predicted increases in depressive symptoms in adolescents over a two-year period (Tak et al., 2017). The impact of depressive symptoms on self-efficacy is equally important. Depressive symptoms can erode one’s sense of self-efficacy through cognitive distortions (Persons et al., 2023), reduced motivation (Watson et al., 2020), and impaired performance in various life domains. This creates a potential feedback loop where lowered self-efficacy and increased depressive symptoms reinforce each other. A study by Beasley (2021) demonstrated that interventions targeting self-efficacy led to significant reductions in depressive symptoms, highlighting the potential for self-efficacy enhancement as a therapeutic approach (Beasley, 2021).

In the context of adolescence, this relationship takes on added complexity due to the unique challenges of this developmental stage. Adolescence is characterized by significant biological, cognitive, and social changes, which can impact both self-efficacy beliefs and vulnerability to depression (Zajkowska et al., 2021). For example, changes in academic demands, peer relationships, and identity formation can all influence an adolescent’s sense of self-efficacy and their risk for depressive symptoms (Cattelino et al., 2021). The intricate relationship between self-efficacy and depressive symptoms in adolescents is not fully understood. While numerous studies have analyzed the impact of depression various aspects of psychological functioning, there is a growing need to investigate how self-efficacy specifically interacts with depressive symptoms during adolescence. Particularly, how the network of beliefs that constitutes self-efficacy is restructured in the presence of depressive symptoms remains an area ripe for exploration. Bringing network analysis into psychological research, particularly in studying self-efficacy, offers a new perspective on understanding its complex components and their interrelations (Borsboom and Cramer, 2013). This approach allows for a more nuanced examination of how different aspects of self-efficacy interact and contribute to overall mental health outcomes, especially in adolescents experiencing depressive symptoms.

### 1.1 Network analysis

Network analysis has emerged as a valuable tool in psychological research (Ruan et al., 2022), offering a novel way to study the complex interrelations of psychological constructs (Borsboom, 2017), including self-efficacy. This analytical approach views psychological phenomena not just as isolated elements but as interdependent components within a network (Ruan et al., 2023a). In the context of self-efficacy, network analysis allows researchers to examine how different aspects of self-efficacy interact with each other and contribute to overall mental health outcomes.

By employing network analysis, researchers can identify the most influential components within the self-efficacy construct and understand how changes in one aspect can impact the entire network (Epskamp et al., 2018). This approach is particularly useful in understanding the multifaceted nature of self-efficacy and its role in complex psychological conditions like depression. For instance, network analysis can reveal how specific beliefs about personal competence in various areas (e.g., social situations, academic performance, personal challenges) interact and collectively influence an individual’s susceptibility to depression.

Moreover, network analysis in self-efficacy research can provide insights into the dynamic nature of psychological constructs. It allows for the examination of how the relationships between different aspects of self-efficacy change over time, especially in response to developmental transitions or therapeutic interventions. This can be particularly insightful in adolescent populations, where rapid developmental changes are a defining characteristic.

The current study aims to fill this gap by examining the self-efficacy networks in adolescents with and without depressive symptoms. By employing network analysis, this research seeks to unravel the complex interplay between various components of self-efficacy and how they are potentially reorganized in the context of depressive symptomatology. The study utilizes robust statistical methods, including the least absolute shrinkage and selection operator (LASSO) technique and network centrality analysis, to construct and compare self-efficacy networks between depressed and healthy control groups (Friedman et al., 2008). This approach allows for a more nuanced understanding of the multifaceted nature of self-efficacy in adolescent depressive symptoms, going beyond traditional unidimensional perspectives.

## 2 Method

### 2.1 Participant

The participants were youth residents of Wenzhou City aged 12–18 selected through a clustered random sampling method. The data were collected as part of a mental health screening program for youth jointly implemented by the Seventh People’s Hospital of Wenzhou City and psychiatric hospitals across six districts of Wenzhou City, including Ouhai, Ruian, Lucheng, Longwan, Yongjia, Yueqing, and Taishun. Participants completed psychological assessments administered by trained researchers and clinicians at local youth psychiatric facilities. Ethical adherence was maintained throughout, conforming to national and institutional human experimentation standards and the Declaration of Helsinki (revised 2008). All procedures involving human patients were approved by IRB in Wenzhou Seventh People’s Hospital (EC-KY-2022048).

### 2.2 Measurements

#### 2.2.1 Demographic information

Demographic information including gender, age, and education level was collected using a self-administered questionnaire. Gender was reported as male, female, or other. Age was reported in years. Education level was reported in terms of highest degree completed, with response options of primary school or below, high school, college and above.

#### 2.2.2 Depressive symptoms

Depressive symptoms were measured using the Patient Health Questionnaire-9 (Kroenke et al., 2001). The PHQ-9 contains 9 items assessing depressive symptoms over the prior 2 weeks and is based on DSM-IV diagnostic criteria. Participants rated symptoms on a 4-point frequency scale from 0 (not at all) to 3 (nearly every day). Responses are summed for a total score ranging from 0 to 27. In line with prior convention (Kroenke et al., 2001), a cutoff score of 10 was used in this study to determine clinical significance, with scores of 10 or greater denoting the depressed group. The PHQ-9 has demonstrated excellent validity and reliability in both clinical and non-clinical populations, with Cronbach’s alpha = 0.90 in this study.

#### 2.2.3 Self-efficacy

Self-efficacy was assessed using the 10-item General Self-Efficacy Scale (GSES) developed by Luszczynska et al. (2005). The GSES is designed to assess an individual’s belief in their ability to respond to novel or difficult situations and deal with any associated obstacles or setbacks. Participants rate items such as “I can always manage to solve difficult problems if I try hard enough” on a 4-point scale from 1 (not at all true) to 4 (exactly true). Scores are summed to create a total self-efficacy score, with higher scores indicating greater perceived self-efficacy. The GSES has frequently been used in the literature and has demonstrated good reliability and validity across cultures and contexts, with Cronbach’s alpha = 0.94 in this study.

### 2.3 Statistics analysis

Descriptive analyses were conducted to report continuous variables as means and standard deviations, and categorical variables as frequencies and percentages. Differences in components were analyzed using independent samples *t*-tests and chi-square analyses. Bivariate correlations were examined using Pearson’s correlation analysis.

The primary objective of the statistical analysis was to construct a self-efficacy measurement network. To this end, we employed the least absolute shrinkage and selection operator (LASSO) technique, a form of regularized regression well-suited for network construction (Epskamp et al., 2018). LASSO aids in identifying the most relevant connections (edges) between the variables (nodes) by penalizing the absolute size of the regression coefficients, thereby reducing overfitting and enhancing model accuracy (Jones et al., 2018). This approach was instrumental in delineating the intricate network of inter-item relationships constituting the self-efficacy construct in our sample.

Following the network construction, we conducted a network centrality analysis. This analysis focused on identifying key nodes within the self-efficacy network that held significant influence or centrality. Centrality metrics such as strength, closeness, betweenness and expected influence were calculated (Borsboom, 2017). These metrics provided insights into the relative importance and influence of specific self-efficacy items within the overall network. Ensuring the robustness and reliability of the network model was imperative. To assess the stability of the network, we employed bootstrapped subsamples to examine the consistency of the edge weights and centrality indices. This involved repeatedly resampling the data and recalculating the network metrics to determine their variability. The Case-Dropping Subset Bootstrap (CS-coefficient) was used to quantify the stability, with higher values indicating greater reliability of the network’s features (Luo et al., 2022). Then a comparative examination of the networks between the depressed and healthy control groups. This was executed to discern any overarching differences in the overall strength and structure of the self-efficacy networks across these groups (van Borkulo et al., 2015). The network comparison was twofold: firstly, we assessed the global strength, a measure of the overall interconnectedness within the network, and secondly, we evaluated the structural invariance using statistical tests (M for structural invariance and S for strength invariance).

Finally, bridge analysis was conducted to further understand the connection between depressive symptoms and self-efficacy. Bridge nodes are nodes that play a crucial role in connecting different clusters or communities within a network. Bridge centrality measure was estimated, which quantifies the extent to which a node connects disparate parts of the network (Jones et al., 2021).

All statistical analyses were performed using R version 4.3.2, employing packages such as “bootnet,” “qgraph” and “NetworkComparisonTest” with a significance level set at *p* < 0.05.

## 3 Result

### 3.1 Descriptive analysis

The sample for this study consisted of 3,654 subjects, with a mean age of 15.13 years (*SD* = 2.06). The subjects comprised 2044 males (55.94%) and 1,610 females. The majority of the subjects (2079, 56.90%) indicated their current academic qualifications were high school. In assessing mental health states with the PHQ-9, 560 subjects met criteria for moderate to severe depressive symptoms (defined as PHQ-9 scores ≥10), representing 15.32% of the total sample. Of these, 373 subjects were classified as having moderate depressive symptoms (PHQ-9: 10–14), 126 as having moderate–severe depressive symptoms (PHQ-9: 15–19), and 61 as having severe depressive symptoms (PHQ-9 ≥ 20). Independent samples t-test and chi-square test revealed statistically significant differences in age (*t* = 3.08, *p* = 0.002), gender (*χ*^2^ = 14.92, *p* = 0.01) and educational levels (*χ*^2^ = 8.59, *p* = 0.01). Self-efficacy was significantly lower among subjects with depressive symptoms relative to the control group (*t* = 17.08, *p* < 0.001). The effect size for this group difference in self-efficacy was large (Cohen’s *d* = 0.78) (Table 1). In addition, bivariate correlation analyses of depressive symptoms and self-efficacy indicated a significant negative correlation (*r* = −0.41, *p* < 0.001), see Table 2.

**Table 1:** Demographic information for depressive symptoms group and healthy control group.

Variables	Depressive symptoms (*n* = 560)		Health control (*n* = 3,094)		*t/χ^2^*	*p*
	*M*/*n*	*SD*/*percent*	*M*/*n*	*SD*/*percent*		
Age	14.88	1.83	15.17	2.09	3.08	0.002
Gender					14.92	0.01
Male	271	48.39%	1773	57.30%		
Female	289	51.61%	1,321	42.70%		
Educational levels					8.59	0.01
Primary School or Below	246	43.92%	1,195	38.62%		
High School	302	53.92%	1777	57.43%		
College and Above	12	2.14%	122	3.94%		
Depressive symptoms	13.94	3.99	3.21	2.97	74.31	< 0.001
Self-efficacy	21.86	6.04	27.29	7.07	17.08	< 0.001

**Table 2:** Matrix of correlations among demographic variables, depressive symptoms, and self-efficacy.

Variables					
1. Age	1				
2. Gender	< 0.001	1			
3. Educational levels	0.86^***^		1		
4. Depressive symptoms	−0.03	−0.11^***^	−0.02	1	
5. Self-efficacy	−0.06^**^	0.15^***^	−0.01	−0.41^***^	1

### 3.2 Self-efficacy measurement network

**Figure 1:**
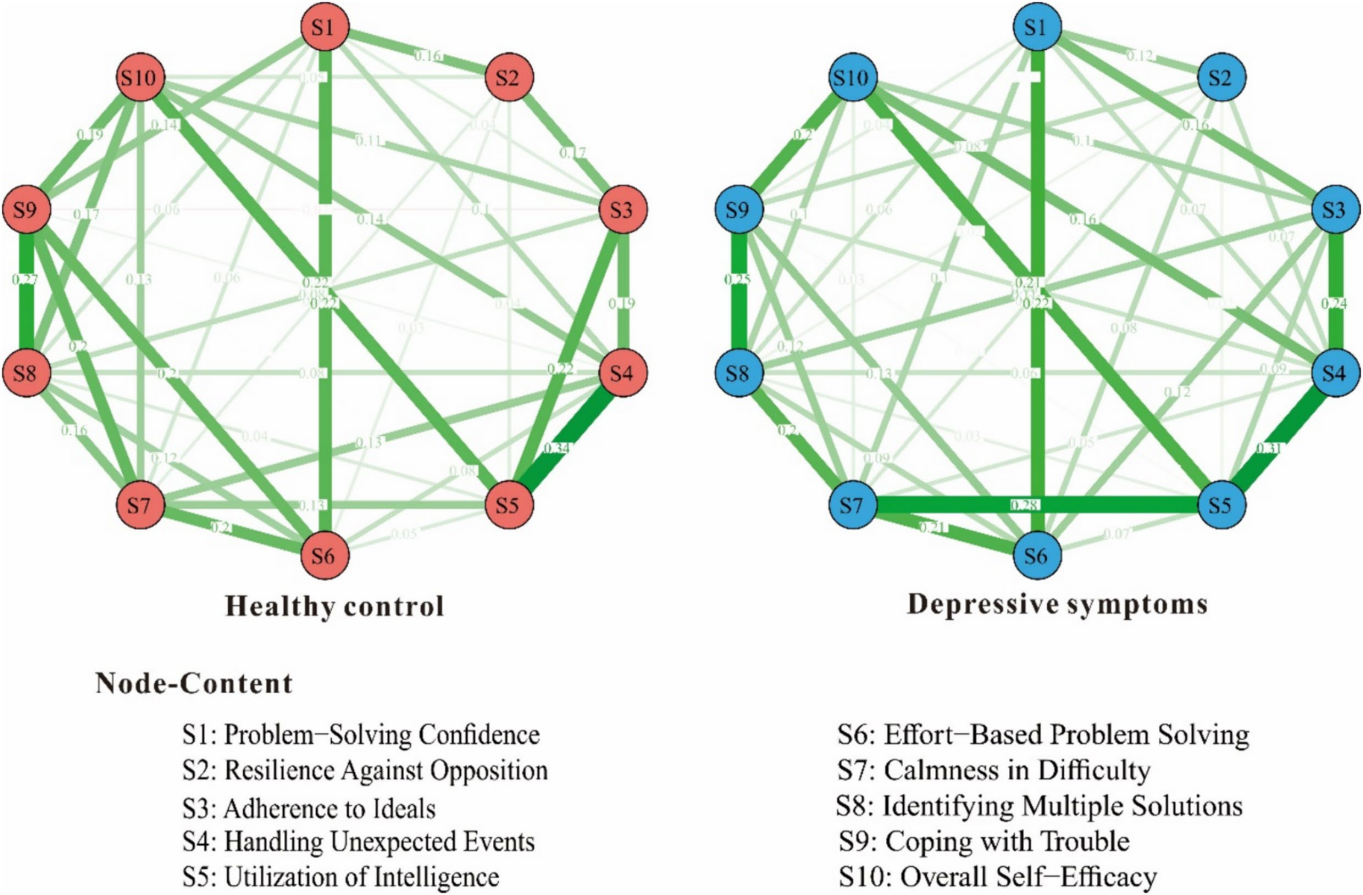
Self-efficacy measurement network in depressive symptoms group and healthy control group.

To elucidate the differences in self-efficacy between the depressive symptoms group (*n* = 560) and healthy control group (*n* = 3,094), separate network models were constructed for each group based on 10 items assessing self-efficacy, refer to Figure 1. In the depressive symptoms group network model, there were 39 non-zero edges out of a possible 45 edges (86.67%). The mean edge weight for the 39 connected items in the depressed group network was 0.096. In the healthy control group network model, 37 non-zero edges were present out of the 45 possible connections between self-efficacy items (82.22%). The average edge weight between the 37 associated items in the control group network model was 0.101.

**Figure 2:**
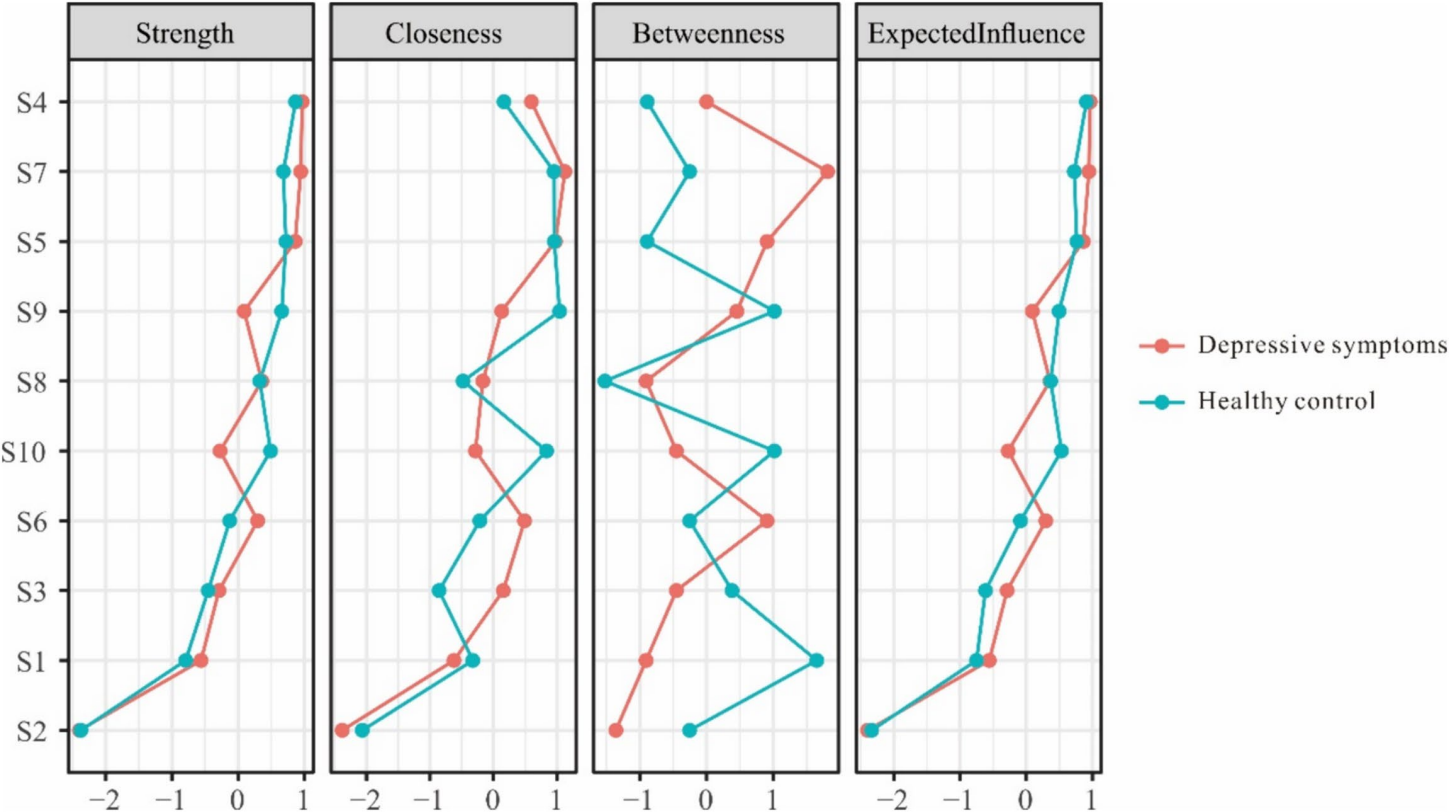
Centrality indices for the nodes of the self-efficacy measurement network.

Upon further examination, the edge weights in the self-efficacy networks were highly consistent between the depressed group and healthy control group. The strongest edge weights in both network models were S4-S5. In terms of centrality indices, the network structures also demonstrated similar patterns across groups. S4 had the highest intensity centrality with strength = 0.96 in depressive symptoms group network model and strength = 0.87 in healthy control group network model, refer to Figure 2.

Tests of network stability were also performed to determine the reliability of edge weights and centrality metrics for each group’s self-efficacy network structure. The results showed good accuracy and stability of edges (CS-coefficient = 0.60/0.67), strength (CS-coefficient = 0.67/0.75), expected influence (CS-coefficient = 0.75/0.75) and closeness (CS-coefficient = 0.60/0.60) in depressive symptoms group and healthy control group. However, low stability for betweenness centrality (CS-coefficient = 0.05/0.05) suggests paths connecting distant nodes may vary across bootstrapped iterations of each network.

### 3.3 Network comparison

To directly test distinctions in network connectivity between the depressive symptoms and healthy control groups, both network invariance and global strength invariance analyses were conducted. The network invariance test yielded a test statistic of *M* = 0.166 and *p* = 0.042. Since the *p*-value falls below the 0.05 significance level, the test indicates that some edge parameters significantly differ between groups. For the global strength invariance test, the depressed group had an overall lower global strength of 4.33 compared to 4.58 for the control group network. The test statistic was *S* = 0.25 and *p* = 0.031. The significant p-value provides further evidence that overall connectivity strength is lower across items in the depressed group’s self-efficacy network. In combination with the observed variability in specific edge weights and centrality patterns reported earlier, these findings from direct group comparison signify that the network structure of self-efficacy perceptions substantively varies based on depressive symptoms status.

Followed edge-weight testing was conducted. The comparative analysis, as reflected in Table 3, unveils significant changes in the connectivity of specific edges within the self-efficacy networks of both groups. Notably, the edge connection between S1 (“Problem-Solving Confidence”) and S3 (“Adherence to Ideals”) was found to be strengthened in the depressive symptoms group, increasing from 0.04 in the healthy control group to 0.16, with a statistically significant (*p* < 0.05). This enhancement indicates an increased association between these items in the context of depressive symptoms. Conversely, the connection between S2 (“Resilience Against Opposition”) and S3 (“Adherence to Ideals”), evident in the healthy control group with a weight of 0.16, was absent in the depressive symptoms group, signifying a loss of this specific linkage (*p* < 0.01). Additionally, new links emerged in the depressed group’s network, which were not present in the healthy controls. For instance, the S2 (“Resilience Against Opposition”) S4 (“Handling Unexpected Events”) and S3 (“Adherence to Ideals”) S6 (“Effort-Based Problem Solving”) connections, represented by edge weights of 0.07 and 0.11 respectively, were not observed in the healthy control group. These new links, with respective *p*-values of 0.02 and 0.002, indicate novel associations unique to the depressive symptoms group’s self-efficacy network. Other notable changes included the weakening of the S3 (“Adherence to Ideals”) S5 (“Utilization of Intelligence”) and S1 (“Problem-Solving Confidence”) S9 (“Coping with Trouble”) connections in the depressive symptoms group, as evidenced by decreased weights and significant *p*-values (0.01 and 0.03, respectively). In contrast, the S5 (“Utilization of Intelligence”) S7 (“Calmness in Difficulty”) connection was considerably strengthened in the depressive symptoms group, moving from 0.13 in the healthy control group to 0.28, with a p-value of 0.01.

**Table 3:** The change of the symptom-connected profile from the health to depressive symptoms.

Edge connection	Healthy control group	Depressive symptoms group	*P-value*	Change
S1–S3	0.04	0.16	0.03	Strengthened
S2–S3	0.16	(--)	0.01	Lost
S2–S4	(--)	0.07	0.02	New link
S3–S5	0.22	0.09	0.01	Weaken
S3–S6	(--)	0.11	0.002	New link
S5–S7	0.13	0.28	0.01	Strengthened
S1–S9	0.14	0.04	0.03	Weaken
S2–S9	(--)	0.08	0.04	New link

### 3.4 Network bridge analysis

**Figure 3:**
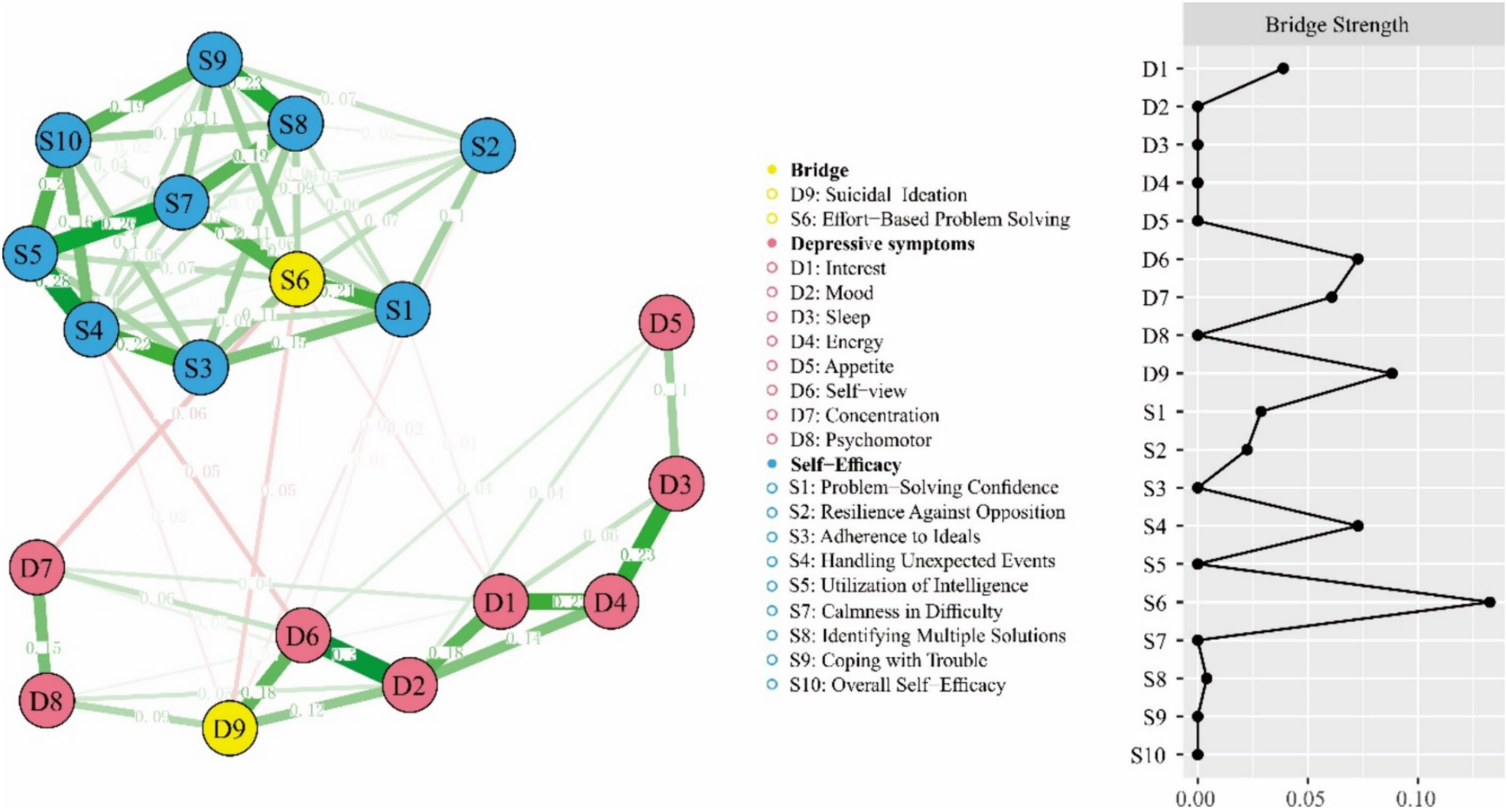
Depressive symptoms and self-efficacy network model with bridge centrality measure.

The bridge analysis was illustrated in Figure 3. The strongest connection was found between S6 (“Effort-Based Problem Solving”) and D7 (“Concentration”) with edge weight = −0.06, followed by the association between S6 (“Effort-Based Problem Solving”) and D9 (“Suicidal Ideation”), with an edge weight of −0.05. S6 (“Effort-Based Problem Solving”) was the self-efficacy node with the most direct connections to the depressive symptom cluster (S6-D1, D7, D9). D9 (“Suicidal Ideation”) emerged as the depressive symptom node with the most direct connections to the self-efficacy symptom cluster (D9-S1, S4, S6,). Bridge centrality measure also identified S6 (“Effort-Based Problem Solving”) and D9 (“Suicidal Ideation”) as the key bridge nodes between self-efficacy and depressive symptoms, with bridge strengths of 0.13 and 0.09, respectively.

## 4 Discussion

The present study’s exploration of self-efficacy network structures in adolescents with and without depressive symptoms reveal critical insights into the multifaceted nature of self-efficacy in the context of mental health. These insights not only challenge conventional views but also enrich our understanding of the complex interplay between cognitive processes and depressive symptomatology.

### 4.1 Reduced global strength in the self-efficacy network of the depressed group

The most salient finding of our study was the altered network structure of self-efficacy in the depressive symptoms group. The depressive symptoms group exhibited a network with reduced global strength, suggesting an overall diminished interconnectedness among self-efficacy items and a more fragmented perception of self-efficacy. This finding aligns with Bandura’s theory, which posits that lowered self-efficacy can negatively impact an individual’s ability to cope with adversity, potentially exacerbating depressive symptoms (Benight and Bandura, 2004). The mindsponge theory offers a novel perspective to interpret this result. Mindsponge theory suggests that individuals with depressive symptoms have a diminished capacity to absorb, process and integrate self-efficacy-related information effectively (Vuong, 2023). In the context of the mind sponge framework, depression can be conceptualized as a state where the brain’s reduced capacity to process and integrate positive self-efficacy information results in a weakened and more disconnected self-efficacy network. Furthermore, the diminished interconnectedness among self-efficacy items reflect the brain’s tendency to focus on and retain negative information in depression, leading to a distorted and compartmentalized self-view (Mennen et al., 2019). This biased information processing, as explained by the mindsponge theory, may exacerbate depressive symptoms by reinforcing a sense of incompetence and helplessness.

### 4.2 Altered self-efficacy network dynamics in depressive symptoms

The strengthening of specific edges, such as between Problem-Solving Confidence (S1) and Adherence to Ideals (S3), highlights important aspects of cognitive functioning in individuals with depressive symptoms. This pattern aligns with Beck’s cognitive model of depression, which posits that individuals with depression tend to develop and maintain negative cognitive biases. Previous research has demonstrated that individuals with depression often exhibit lower problem-solving confidence. Lower confidence in one’s problem-solving abilities can lead to feelings of helplessness and hopelessness, core features of depressive symptomatology (López et al., 2020). Furthermore, adherence to unrealistic ideals, or perfectionism, has been identified as a significant risk factor for depression (Bull et al., 2022). Individuals who rigidly adhere to high standards may experience chronic dissatisfaction and self-criticism, contributing to the maintenance of depressive symptoms (Werner et al., 2019). Similarly, the emergence of new links in the depressive symptoms group, like the connection between Resilience Against Opposition (S2) and Handling Unexpected Events (S4), indicate adaptive or maladaptive shifts in self-efficacy dynamics in response to depressive symptoms. It suggests a potential reorganization of cognitive resources or coping strategies in the face of psychological distress, a concept not extensively explored in current adolescent depression research (Li and Kwok, 2023). On the other hand, the weakening of connections, such as between Adherence to Ideals (S3) and Utilization of Intelligence (S5), and between Problem-Solving Confidence (S1) and Coping with Trouble (S9), in the depressive symptoms group points to a potential disruption in integrating cognitive and problem-solving aspects of self-efficacy (Villalobos et al., 2021). Depression impairs cognitive functions related to problem-solving and executive function, including working memory and cognitive flexibility (Pan et al., 2019).

The observed pattern of strengthened and weakened connections within the self-efficacy network among depressed adolescents can be interpreted through the cognitive vulnerability-stress model. This model suggests that pre-existing cognitive vulnerabilities, when combined with stressors, heighten the risk for developing depressive symptoms (Gladstone et al., 2022). The strengthened connections in our study might indicate an overactive self-regulatory response. Conversely, the weakened connections might reflect areas of cognitive avoidance, a common feature in depressive cognition (Mellick et al., 2019). Moreover, these findings can be contextualized within the neurodevelopmental changes characteristic of adolescence (Pfeifer and Allen, 2021). The ongoing maturation of the prefrontal cortex, pivotal for self-regulation and executive functioning, could be influencing the reorganization of self-efficacy networks, particularly in the presence of depressive symptoms. These neurodevelopmental transformations might underlie the shifts in how adolescents process self-related information, especially in the presence of depressive symptoms. For instance, research shows that depressive symptoms can disrupt the typical neurodevelopmental trajectory of the prefrontal cortex (Whitfield-Gabrieli et al., 2020).

### 4.3 Bridging self-efficacy and depressive symptoms

The identification of effort-based problem-solving and suicidal ideation as the strongest bridge in our network analysis has significant implications for understanding the complex relationship between cognitive processes and depressive symptoms. This finding is consistent with a growing body of literature that emphasizes the central role of problem-solving deficits in the onset, maintenance, and recurrence of depression (Smith et al., 2019; Jones-Bitton et al., 2020). For example, a computer-based simulation experiment conducted by Kipman et al. revealed that individuals with depressive symptoms are associated with lower problem-solving abilities compared to healthy controls (Kipman et al., 2022). Individuals who experience suicidal thoughts also exhibit impairments in their ability to generate effective solutions to problems, leading to a sense of hopelessness and helplessness that can perpetuate depressive symptoms. Labelle et al. found that adolescents with suicidal ideation displayed significantly worse problem-solving skills than those without suicidal thoughts, highlighting the link between these cognitive deficits and suicidal tendencies (Labelle et al., 2013).

Furthermore, the identification of effort-based problem-solving as a bridge node highlights its potential as a target for therapeutic interventions designed to reduce suicidal ideation and improve mental health outcomes (Jiang et al., 2021). Zhang et al. demonstrated that changes in problem-solving appraisal following cognitive therapy were associated with reductions in suicidal ideation, supporting the notion that targeting this cognitive process can lead to meaningful improvements in mental health (Zhang et al., 2021). Cognitive-behavioral therapy (CBT) has been shown to be particularly effective in addressing problem-solving deficits and suicidal ideation (Sander et al., 2023; Xu et al., 2023). A randomized controlled trial by Sinyor et al. found that CBT significantly reduced the risk of future suicide attempts in individuals with a history of suicidal behavior, emphasizing the efficacy of this therapeutic approach (Sinyor et al., 2020). By teaching individuals adaptive problem-solving skills and challenging negative cognitive biases, CBT can help break the cycle of hopelessness and suicidal thoughts. Becker-Weidman et al. (2010) reported that adolescents treated for depression with CBT showed significant improvements in social problem-solving abilities, further supporting the role of CBT in addressing these cognitive deficits (Becker-Weidman et al., 2010).

## 5 Conclusion

This study aimed to analyze the relationship between self-efficacy and depressive symptoms in adolescents using network analysis. Our findings indicate significant differences in self-efficacy networks between adolescents with and without depressive symptoms. Specifically, adolescents with depressive symptoms exhibited a network with reduced global strength, suggesting diminished interconnectedness among self-efficacy items. This diminished integration underscores the complex interplay between self-efficacy and depressive symptoms, challenging the traditional unidimensional view of self-efficacy. Additionally, effort-based problem-solving and suicidal ideation were identified as key bridge nodes, suggesting that interventions aimed at enhancing problem-solving skills and addressing suicidal thoughts could be particularly effective. These findings provide a foundation for developing targeted therapeutic strategies to improve mental health outcomes in adolescents. In conclusion, this study provides valuable insights into the relationship between self-efficacy and depressive symptoms in adolescents. By understanding the intricate network of self-efficacy, we can better support adolescents in overcoming mental health challenges and promote their psychological well-being.

## 6 Limitations

In our study exploring self-efficacy networks in adolescents with depressive symptoms, several limitations must be acknowledged. The generalizability of our findings is constrained by the limited diversity in our sample, highlighting the need for future research with more varied demographic groups (Roberson et al., 2017). The cross-sectional design precludes causal inferences, underscoring the importance of longitudinal studies to understand the temporal evolution of these networks (Prati and Mancini, 2021; Ruan et al., 2023b). Reliance on self-report measures may introduce biases, suggesting a need for more objective or multi-informant approaches in subsequent research (Giromini et al., 2022). Additionally, the potential neurodevelopmental influences on self-efficacy networks were not directly assessed, indicating an opportunity for future studies to integrate neuroimaging or psychophysiological measures (Schmälzle et al., 2021). These limitations open several avenues for future research, particularly in tailoring intervention strategies, exploring the mechanisms behind network alterations, and examining the role of environmental and social factors in shaping self-efficacy in the context of adolescent depressive symptoms.

## Data Availability

The raw data supporting the conclusions of this article will be made available by the authors, without undue reservation.
